# Adverse Effects of Black Carbon (BC) Exposure during Pregnancy on Maternal and Fetal Health: A Contemporary Review

**DOI:** 10.3390/toxics10120779

**Published:** 2022-12-13

**Authors:** Viktoriia Goriainova, Christina Awada, Florence Opoku, Judith T. Zelikoff

**Affiliations:** Division of Environmental Medicine, Grossman School of Medicine, New York University, New York, NY 10010, USA

**Keywords:** air pollution, black carbon, pregnancy, placenta, respiratory disease, cardiometabolic disease, inflammation, low birth weight, preterm birth, gestational diabetes mellitus

## Abstract

Black carbon (BC) is a major component of ambient particulate matter (PM), one of the six Environmental Protection Agency (EPA) Criteria air pollutants. The majority of research on the adverse effects of BC exposure so far has been focused on respiratory and cardiovascular systems in children. Few studies have also explored whether prenatal BC exposure affects the fetus, the placenta and/or the course of pregnancy itself. Thus, this contemporary review seeks to elucidate state-of-the-art research on this understudied topic. Epidemiological studies have shown a correlation between BC and a variety of adverse effects on fetal health, including low birth weight for gestational age and increased risk of preterm birth, as well as cardiometabolic and respiratory system complications following maternal exposure during pregnancy. There is epidemiological evidence suggesting that BC exposure increases the risk of gestational diabetes mellitus, as well as other maternal health issues, such as pregnancy loss, all of which need to be more thoroughly investigated. Adverse placental effects from BC exposure include inflammatory responses, interference with placental iodine uptake, and expression of DNA repair and tumor suppressor genes. Taking into account the differences in BC exposure around the world, as well as interracial disparities and the need to better understand the underlying mechanisms of the health effects associated with prenatal exposure, toxicological research examining the effects of early life exposure to BC is needed.

## 1. Introduction

Black carbon (BC), also called elemental carbon as it consists of pure carbon bound together in several forms, is a major component of fine particulate matter (PM) with an aerodynamic diameter less than or equal to 2.5 μm (PM_2.5_). Black carbon has not been well defined as it is often confused with soot, which consists of graphite-like carbon layers. The definition of BC, on the other hand, should be restricted to carbonaceous matter of uncertain character that absorbs and reflects light [1]. Black carbon is also continuously confused with carbon black (CB). However, these terms are not interchangeable, as carbon black is “a manufactured product with well-controlled properties whereas BC is an undesired, incomplete-combustion byproduct with diverse properties” [2]; BC particles are formed during incomplete combustion of fossil fuel, biofuel, and biomass, and thus heavy emissions arise from household devices, such as cookstoves and fireplaces. Forest/wildfires have also proven to be a major source of BC [3]. Apart from those sources, both naturally occurring and anthropogenic soot consists primarily of BC particles. More than half of the anthropogenic emissions of BC into the Earth’s atmosphere comes from Asia, with China being the biggest contributor [4]. Some predictions concerning the growth of BC emissions suggest rates up to 2183 Gg/yr by the year 2050.

Currently, there are no US federal standards for BC. The U.S. Environmental Protection Agency (EPA) provides a primary (health-based) annual standard for PM_2.5_ at 12.0 µg/m^3^ and secondary (welfare-based) annual standard for PM_2.5_ at 15.0 µg/m^3^; PM_2.5_ absorbance often serves as a proxy for BC exposure [5]. However, no regulations currently exist regarding the composition of PM_2.5_, even though health effects can vary widely depending on the source and percentages of individual components [6]. Black carbon exposure varies greatly between different regions of the world, providing the potential for health disparities. For example, cookstoves that use biomass as a fuel are still used by 60% of all households in India [7], where mostly women use them to cook under unventilated conditions, which makes them the primary household members exposed to BC. Under such conditions, pregnant women and their fetus are at even greater risk as BC has been shown to pass the placental barrier [8].

A variety of organ systems in both children and adults can be adversely impacted by the inhalation of BC particles. Both short-term and long-term BC exposure can lead to increased hospitalizations due to respiratory and cardiovascular morbidity [9]. Underlying diseases, such as coronary artery disease and diabetes, can exacerbate development of subclinical cardiovascular morbidity resulting from BC exposure [10]. It has also been shown that exposure to BC in childhood is associated with a higher risk of asthma attacks [11] and decreased cognitive function, including altered behavioral development [12]. However, it is less clear how prenatal exposure to BC might affect fetal health and pregnancy itself. In this contemporary review, we will examine how inhalation exposure to BC not only can affect the mother’s health during pregnancy, but also pregnancy outcomes and fetal health (Table 1).

The studies discussed in this review were selected by searching PubMed and Google Scholar using the following terms: “black carbon” and “routes of exposure” OR “fetus” OR “infants” OR “children” OR “placenta” OR “maternal health” OR “pregnancy loss” OR “diabetes” and were published between 2008–2022. A total of 42 studies pertaining to the topic were found, and 11 were excluded as only an abstract was openly accessible, therefore 31 studies were considered. This review primarily focuses on short-term effects of prenatal BC exposure, as the majority of published data are focused on such outcomes; there are currently very few studies that focus on long-term or persistent effects.

## 2. Epidemiological Studies

### 2.1. Impact of BC on Fetal and Child Health

#### 2.1.1. Gestational Age and Low Birth Weight

Fetal exposure is determined in part by whether substances have the ability to cross the placenta, i.e., the maternal-fetal barrier [44,45]. The primary route of exposure to BC is inhalation. BC particles are easily distributed throughout the body due to their small size (0.01–1 µm) and large surface area making them highly respirable and able to penetrate deep into the lungs [46]. In 2019, the first evidence emerged that inhaled BC particles have the ability to translocate from the mother’s lungs, through the placenta to the fetus, where they can accumulate on the fetal side of the placenta [8]. Fetal exposure to BC varies depending on the mother’s exposure to BC and stage of fetal development at the time of exposure. The thickness and permeability of the placenta changes as an embryo develops into a fetus, with the placenta being thicker and less penetrable early in pregnancy and gradually becoming thinner and more permeable during the last three months [47,48]. The thinning of the placenta can lead to increased risk for adverse birth outcomes, if the fetus is exposed to BC later in pregnancy [49].

Gestational age and birth weight are well-known predictors for overall neonatal health. Low birth weight is linked to increased neonatal mortality and morbidities, including delayed visual and cognitive development, emotional-behavioral defects during childhood, and increased risk of various diseases in adulthood such as hypertension, dyslipidemia, and chronic kidney disease [50,51,52,53]. Many epidemiological studies have linked ambient air pollution to reduced fetal growth, low birth weight, and premature birth. In a cohort study conducted in Greater Boston, infants who were exposed during the third trimester of pregnancy to the highest quartile of BC (vs. lowest quartile) had birth weight-for-gestational age z-score of −0.17 when adjusted for the covariates with a 95% confidence interval (CI) = −0.29 to −0.05 (−0.31 before adjusting with CI = −0.43 to −0.19), where the exposure was positively correlated to mothers living close to major roadways [13]. A similar cohort study investigated birth outcomes affected by traffic-related air pollution exposure in Vancouver, British Columbia [14]. The authors demonstrated that small-for-gestational age birth and low birth weight risk were increased in infants prenatally exposed to BC, PM_10_, PM_2.5_, carbon monoxide (CO), and nitrogen oxide (NOx). Namely, the odds ratio (OR) of small for gestational age birth was 1.02 for PM_2.5_ (95% CI = 1.00–1.03) and 1.01 for BC (95% CI = 0.99–1.03) after adjusting for covariates; the OR for low birth weight was 1.03 (95% CI = 0.99–1.07) and 1.00 (95% CI = 0.95–1.07), respectively. These results were based on approximating exposures with land use regression models (LURs). The results based on the proximity to major traffic were much more robust: mothers residing within 50 m of a highway had a 26% increase in risk of small for gestational age birth (95% CI = 1.07 to 1.49), and an 11% increase for risk of low birth weight in full-term infants (95% CI = 1.01 to 1.23). A study from 2018 evaluated interactions between early pregnancy exposure to traffic pollution by BC output, cigarette smoke, and per- and polyfluoroalkyl substances (PFAS) and birth weight-for-gestational-age; the authors estimated a 0.08 reduction (95% CI = −0.15 to −0.01) in birth weight-for-gestational age for each interquartile range increased increment in BC exposure during the first trimester [15]. In a study published in 2021, authors observed patterns between prenatal exposure to PM_2.5_, BC, NH_4_^+^, NO_3_^2−^, and SO_4_^2−^ and decreased weight-for-length z-score (WLZ) and weight-for-age z-score (WAZ) in schoolchildren aged 1-, 4-, and 6 years of age [16]. In particular, at 6 years old, WAZ in boys decreased by −1.081 for the 2nd trimester exposure (95% CI = −1.915 to −0.24) and by −0.855 (95% CI = −1.587 to −0.124) during the 3rd trimester BC exposure. WLZ/BMIz significantly decreased in both boys and girls as measured at 4- and 6 years of age, and revealed an association with exposure throughout pregnancy. In another recent study [17], it was calculated that throughout pregnancy the average birth weight drops by 17 g for every 0.14 µg/m^3^ increase in BC (95% CI = 15.4 to 18.6) after exposure in the time period of 0–30 days before delivery. The effects of prenatal BC exposure on umbilical cord leukocytes, placenta, and maternal leukocytes’ telomere length, a biomarker of cellular aging, were also recently evaluated [18]. This study determined that for each interquartile range increase in BC exposure in the third trimester, the telomere length in umbilical cord leukocytes decreased by 19% (95% CI = −29 to −6). Interestingly enough, BC exposure in the second trimester had the opposite effect. In this case, telomere length increased by 22% (95% CI = 2 to 46) for each interquartile range increase in BC; placenta and maternal blood cells were not affected by such exposure. The increase in leukocyte telomere length observed after exposure to BC in the second trimester was explained by the authors, in part by inflammatory responses which caused the neutrophil faction to increase in number; enhanced neutrophil numbers have been shown to lead to increased telomere length [54]. Such an inflammatory reaction can also cause T- and B-lymphocytes to proliferate at a higher rate, which in turn can increase the length of an average telomere.

#### 2.1.2. Preterm Birth

Studies have examined the associations between air pollution exposure among pregnant women and preterm birth risk, with some even considering racial disparities. In particular, one group of researchers calculated that the risk of preterm birth in Black women exposed to 0.7 µg BC/m^3^ vs. Black women exposed to 0.14 µg/m^3^ BC was 6.8% higher (95% CI = 0.1 to 13.5) and for Latinx women, the risk was 2.1% higher (95% CI = −1.1 to 5.2) [19]. A study in Rhode Island on preterm birth associated with air pollution exposure used two different exposure assessment approaches and arrived at conflicting conclusions: no association between preterm birth OR and BC exposure was observed as estimated via LUR models, while monitored BC exposure was strongly correlated with preterm birth risk (OR = 1.21, 95% CI = 1.05 to 1.39) [20]. The association between BC exposure and small for gestational age and low birthweight outcomes were inconsistent between different exposure assessment models, as well as between different trimesters. In contrast, a case–control study conducted in twins demonstrated no association between BC exposure and preterm birth; the average BC exposure during pregnancy in the preterm birth case was 3.59 μg/m^3^ (95% CI = 3.29 to 3.77) vs. 3.58 μg/m^3^ (95% CI = 3.35 to 3.74) for the non-preterm birth [21]. An additionally investigated adverse birth outcome is premature rupture of membranes (PROM). In a 2020 study, the authors investigated whether air pollution during the 1st trimester was associated with PROM [22]. The adjusted odds ratio (aOR) of PROM in this case was 1.05 (95% CI = 1.01 to 1.09) per each 1 μg/m^3^ of BC exposure during the 1st trimester. These latter findings need to be reproduced for definitive conclusions, and other adverse birth outcomes, such as stillbirth and miscarriage, should be investigated to fill in those gaps in knowledge.

#### 2.1.3. Adverse Neurological Health Effects

There is increasing evidence linking traffic-related pollution to cognitive, behavioral, and neurodevelopmental problems in childhood. In a cohort study conducted in eastern Massachusetts, children whose mothers lived less than 50 m from a major roadway during pregnancy had a 3.8 reduction in verbal IQ (95% CI = −8.2 to 0.6), 7.5 reduction in nonverbal IQ (95% CI = −13.1 to −1.9), and a 5.3 reduction in visual motor skills (95% CI = −11 to 14) compared to children whose mothers lived more than 200 m away from major roadways during pregnancy [23]. However, full adjustment for BC exposure during the 3rd trimester failed to show a significant difference. Interestingly, another group of researchers analyzing the same cohort found that 3rd trimester BC exposure was associated with lower scores on Behavior Rating Inventory of Executive Function (−1.2 on Metacognition Index, 95% CI = −2.2 to −0.2), and lower scores on the Strengths and Difficulties Questionnaire (−0.9 on total difficulties, 95% CI = −1.4 to −0.4), which suggests enhanced executive function [24].

A cohort study from Boston, MA investigated whether exposure to traffic-related BC impacted neurodevelopmental abilities in children exposed in utero [25] by measuring neurodevelopment 6 years later via Wide Range Assessment for Memory and Learning (WRAML2). The study revealed differences in learning and memory performance in male and female children exposed to the same level of BC; for each interquartile increase in BC exposure, Attention Concentration Index (ACI) in boys was lowered by −6.03 (95% CI = −12.8 to 0.76), whereas ACI in girls was reduced by only by −1.86 (95% CI = −8.4 to 4.6). Taken together, these findings emphasize the importance of analyzing cognitive endpoints in the context of prenatal BC exposure.

#### 2.1.4. Adverse Respiratory Health Effects

In contrast to the plethora of studies investigating the effects of early life air pollution exposure, as an entire entity on child’s respiratory health, very few focused on the consequences of prenatal BC exposure, alone. Clark et al., (2010) conducted a case–control study of children with and without asthma, where they analyzed the air pollution levels to which children were exposed to in utero and during the first year of life [26]. Prenatal exposure to BC, measured as 10^−5^/m increase in filter-based light absorbance, was higher in asthmatic children than controls (1.37 ± 0.66 vs. 1.34 ± 0.65, respectively). In a study released in July of 2022, authors reported that maternal exposure to air pollution during pregnancy increased the risk in kindergarteners of developing hay fever and allergic rhinitis, a chronic inflammatory disease affecting nasal pathways [27]. Each IQR increase in maternal exposure to BC during pregnancy was associated with an increased prevalence ratio of doctor-diagnosed allergic rhinitis (1.42; 95% CI = 1.21 to 1.66), while prevalence ratio of current hay fever was increased by 1.58 (95% CI = 1.31 to 1.90). Another study examined the effects of prenatal exposure to air pollution on wheezing patterns in children as young as 2 years of age [28]. The authors observed a statistically significant associations between high prenatal BC exposure and wheeze (OR = 1.84, 95% CI = 1.08 to 3.12). More studies focusing on prenatal exposure to BC are warranted to confirm these findings.

#### 2.1.5. Adverse Cardiometabolic Health Effects

There are multiple epidemiological studies linking BC exposure to the development or progression of chronic non-communicable diseases, such as cardiovascular disease, diabetes, and osteoporosis [55,56,57,58,59]. For example, a study from Belgium investigated the effect of pre- and postnatal exposure to BC, PM_2.5_, PM_10_, BC, and NO_2_ on development of heat-related skin hyperemia, which was used as a biomarker for microvasculature [29]. Skin microvascular blood tests were performed on children between 4 and 6 years-of-age. Results revealed that an IQR increment in BC exposure during the third trimester of pregnancy was associated with an 11.5% decrease in skin hyperemia (95% CI = −20.1 to −1.9). There is also emerging evidence that BC exposure stimulates a hypertension-like response in utero. In a study using a Boston-area cohort, authors reported that prenatal exposure to ambient PM_2.5_ and BC averaged over 2 to 90 days before birth was associated with a 1.00 mmHg increase in newborn systolic blood pressure (SBP) for each 0.32 μg/m^3^ increase in BC (95% CI = 0.1 to 1.8); in contrast, the same study demonstrated that a 13.5 ppb increase in ozone was associated with a –2.3 mmHg drop in neonatal SBP (95% CI = −4.4 to −0.2) [30]. In the Environmental Influence on Ageing in Early Life (ENVIRONAGE) study, a birth cohort study conducted in Belgium between 2010–2013, authors associated a 1.4 mmHg increase in newborn systolic blood pressure (95% CI = −0.3 to 3.1) and a 1.10 mmHg increase in diastolic blood pressure (95% CI = −0.5 to 2.7) for each 0.5 μg/m^3^ increase in BC exposure during the last 4 months of pregnancy [31]. Another study demonstrated that proximity to a major roadway, and exposure levels of BC and PM_2.5_ at the time of delivery are correlated with cardiometabolic health in infants [32]. The authors determined that higher BC exposure during the 3rd gestational trimester (mean BC concentration of 0.7 µg/m^3^) was correlated with higher leptin concentrations in both early and mid-childhood, as well as higher insulin resistance and fat mass in mid-childhood. Total fat mass of children whose mothers lived less than 50 m from a major roadway at the time of delivery was on average 2.1 kg higher (95% CI = 0.8 to 3.5) than that of children whose mothers lived farther than 200 m away. Interestingly, for each interquartile increment in BC exposure during the 1st and 2nd trimester, insulin resistance decreased by 17.1% (95% CI = −27.6 to −5.2). However, the authors discarded these findings due to inconsistency. Further research examining the effects of BC on cardiovascular and metabolic disease in children is essential.

### 2.2. Impact of BC on Maternal Health

#### 2.2.1. Gestational Diabetes and Type 2 Diabetes

Gestational diabetes mellitus (GDM) is a diabetes diagnosis usually made in the second or third trimester of pregnancy, in the absence of diabetes prior to gestation [60]. As the rate of obesity has increased across the US, so has the prevalence of GDM; prevalence of GDM per 100 people has increased from 4.6 (95% CI = 4.1 to 5.1) in 2006 to 8.2 (95% CI = 7.5 to 8.9) in 2016 with a relatively increased rate of 78% [61]. Both mother and fetus tend to be at a higher risk for adverse health outcomes when the pregnancy is complicated by GDM. Metabolic syndrome, cardiovascular disease, and type 2 diabetes are more likely to develop in mothers with GDM [62]. Studies suggest that women with GDM have a 9.51 times higher risk of progression to type 2 diabetes mellitus (T2DM) throughout their life (95% CI = 7.14 to 12.67) [63]. Both GDM and T2DM are mechanistically similar via changes in insulin resistance and beta-cell dysfunction. The maternal risk factors for GDM have been identified as age, ethnicity, genetic susceptibility, family history of diabetes, obesity, and hypertension [64]. In addition to these well-established risk factors, there is growing evidence that environmental pollutants, such as BC and ambient PM, may be a significant risk factor for GDM. Recent studies in both humans and animals shed light on this possible association. Previous studies have suggested that outdoor air pollution is associated with type 2 diabetes via multiple pathways, such as systemic inflammation, oxidative stress and endothelial dysfunction [65]. Combined Geophysical-Statistical Methods has been used to estimate the average individual exposure concentrations for various air pollutants in the 1st or 2nd, or 1st and 2nd trimesters [33]. The investigators found that exposure to BC was associated with an increased risk of GDM as a result of air pollution exposure in the 2nd trimester of pregnancy (aOR = 1.15 per each IQR increase in BC; 95% CI = 1.07 to 1.25). However, another study using data gathered in Boston (MA) revealed that BC, PM_2.5_ exposure, and traffic density during pregnancy were associated with impaired glucose tolerance (IGT), but not GDM; women exposed to the highest quartile of BC vs. lowest were at a greater risk of developing IGT (OR = 2.87; 95% CI = 0.93 to 8.83) [34]. Prevalence of IGT was increased in the highest PM_2.5_ (OR = 2.63, 95%CI = 1.15 to 6.01) exposure group and those associated with traffic density (OR = 2.66, 95% CI = 1.24 to 5.71). Unfortunately, there are limited studies evaluating the effects of air pollutants on glucose metabolism in pregnancy, specifically in relationship to BC. Most toxicological studies have concentrated on the effects of early life BC exposure rather than maternal exposure during pregnancy. Future epidemiological and toxicological studies are needed to investigate the consequences of BC on both the mother and offspring.

#### 2.2.2. Pregnancy Complications

Current research suggests that air pollution exposure increases the risk of pregnancy complications. A Swedish study has shown that BC, PM_2.5_, PM_10_, and NO_X_ exposure during pregnancy increases the risk of preeclampsia [35]. Preeclampsia cases in the highest vs. lowest quartile of BC exposure during the 3rd trimester had an OR of 1.35 (95% CI = 1.11 to 1.63). Interestingly, preeclampsia cases accompanied by small for gestational age birth had a higher OR of 3.48 for exposure to highest vs. lowest quartile of BC (95% CI = 1.67 to 7.27). Albeit the only study of its kind, it offers robust proof of adverse effects of air pollution on the course of pregnancy.

Another potential birth complication recently investigated is BC-associated pregnancy loss. For example, Massachusetts General Hospital Fertility Center patients were recruited to investigate the association between the time of pregnancy loss and chronic and acute exposure to air pollution among women who were using assisted reproductive technologies (ART) [36]. The authors estimated the daily BC exposure levels (among other pollutants) in relation to the first positive Human Chorionic Gonadotropin (hCG) test until the day of failure or live birth. Their findings suggest that acute and chronic exposure to BC were not associated with pregnancy loss, whereas elevated exposure to NO_2_ revealed an association with increased risk of pregnancy loss 30 days after a positive hCG test [hazards ratio (HR) = 1.34, 95% CI = 1.13 to 1.58]. Increased biological stress during pregnancy can also lead to pregnancy loss. In a recent Belgian study [37], hair samples were collected from pregnant women at the end of the second trimester and after delivery to assess 2nd and 3rd trimester hair cortisol concentrations. Results revealed a 1.76-fold increase in three-month mean residential BC concentrations associated with 1.54-fold increase in third trimester hair cortisol concentrations. The lack of toxicology research specifically investigating the effects of BC in pregnant animals to address multiple unanswered questions is an important research gap that could move the field of BC health research forward.

### 2.3. Impact of BC on the Placenta

Exposure to BC leads to the production of reactive oxygen species (ROS) [66]. The reactions begin with excess formation of ROS leading to 3-nitrotyrosine (3-NTp) production, which is a well-known biomarker of oxidative stress and inflammation [67,68]. It has been shown that 3-NTp formation in the placenta is associated with preeclampsia, GDM, and other high-risk pregnancy conditions [69,70]. Data from ENVIRONAGE Study was used to analyze associations between air pollution exposure and 3-NTp levels in placenta [38]. The investigators determined that exposure estimates to both PM_2.5_ and BC were strongly correlated with placental 3-NTp levels; in particular, it was observed that for each interquartile-range increment of 0.36 µg/m^3^ in BC exposure, placental 3-NTp levels increased by 13.9% (95% CI = −0.21 to 29.9). The effect was mainly attributed to exposure in the first gestational trimester. A toxicological study conducted with rats confirmed that BC exposure induces inflammation and oxidative stress in placenta [39]. In this case, rats were exposed to BC from 6 to 17 days of gestation, and the results demonstrated that the length and dose of exposure to BC was positively correlated with the amount of Hofbauer cells and the expression of NF-κB in the placenta. Findings from these studies suggest that BC exposure is associated with placental-induced nitrosative and oxidative stress, characterized by the excess production of reactive nitrogen and oxygen species.

Another group of researchers, using data from the ENVIRONAGE study, evaluated the correlation between PM_2.5_ and BC exposure and placental iodine uptake [40]. An adequate amount of iodine in the body is required for proper thyroid hormone production, and subsequently, for brain development and growth of the fetus. During pregnancy, iodine is stored in the placenta [71]. Therefore, the amount of placental iodine after birth can be used to evaluate adequacy of iodine supply to the fetus during gestation and the subsequent production of thyroid hormones [72]. The authors calculated that there was a 0.67 µg/kg increase in placental iodine concentration for each 5 µg/m^3^ increment in PM_2.5_ exposure in the second trimester (95% CI = 0.01 to 1.3). PM_2.5_ exposure in the third trimester, however, had an inverse effect on iodine uptake and was associated with a decrease of 0.84 µg/kg in placental iodine concentration for each 5 µg/m^3^ increment in PM_2.5_ (95% CI = −1.54 to −0.13). The same trend was observed with BC and NO_2_ exposure; conversely, the placental iodine levels in this case did not differ significantly between women who had experienced a change in the exposure and those who did not. The increased iodine concentrations after PM_2.5_ exposure in the second trimester were borderline significant when compared to the iodine concentrations in women who had not experienced an increased PM_2.5_ exposure. These observations support previous findings of a correlation between PM_2.5_ exposure in the second trimester and neonatal T4 levels [41]. In the same study [40], it was determined that a 2-standard deviations increase in PM_2.5_ led to a 1.2 μg/dL increase in newborn total thyroxine level (95% CI = 0.5–1.8 μg/dL). Though not directly related to BC levels, the decreased iodine uptake related to PM_2.5_ exposure in the third trimester is indicative of the adverse effects of particulate air pollution, possibly associated with BC on cognitive development.

The ENVIRONAGE cohort study was also used to investigate how BC exposure affects the function of a number of DNA repair and tumor suppressor genes [42]. The researchers noted that the *Alu* mutation rate, which is a marker for overall DNA mutation, was increased by 1.97% for each 0.36 μg/m^3^ interquartile range increment in BC exposure. The same increment was associated with a 9.16% increase in *APEX1* methylation and a 27.56% increase in *ERCC4* methylation, which are genes responsible for DNA repair. These results suggest that exposure to BC decreases the capability of DNA to repair and sustain environmental insults. In addition, another group of investigators established a strong association between BC exposure and expression of one of the placental imprinted genes associated with birthweight [43]. In this case, an interquartile increase of 0.14 μg/m^3^ in BC exposure was associated with a 6.3% decrease in the expression of *BLCAP*, a gene linked to reduced fetal growth [73]. The researchers also observed significant decreases in *ABCA1* expression, which suggests that BC exposure interferes with cholesterol transport between mother and fetus. This finding supports the previous observations of metabolic health changes in infants whose mothers were exposed to BC at the time of delivery [32]. Exposure to PM_2.5_ in this study was also correlated with seven other genes associated with birthweight. Thus, the aforementioned studies provide insight into the mechanisms by which air pollution, particularly BC could affect birthweight and cardiometabolic health.

## 3. Conclusions

Black carbon is a ubiquitous air pollutant which forms a large portion of PM_2.5_. This contemporary review provides an overview of the effects of BC on pregnancy, namely the mother, fetus, and placenta. The research concerning the effects of BC exposure in children is abundant, however, few studies have focused on the effects of prenatal exposure. Adverse health effects of BC exposure have been shown on the respiratory and cardiometabolic system of children, even if the exposure occurred in utero, as BC can be transported to the fetus via the placenta. Studies have also noted that maternal exposure during pregnancy to BC increases the risk of preeclampsia, preterm birth, low birth weight and small weight-for-gestational-age. Biological aging and disease susceptibility may also be affected by BC, as evidenced by telomere shortening and the silencing of DNA repair and tumor suppressor genes.

Black carbon has been proven to produce oxidative stress and inflammation of the placental tissue, which could be an underlying cause for its adverse effects on the course of pregnancy and fetal health. Placental inflammation is often related to preterm birth, low birth weight, and preeclampsia [74,75,76]. In addition, inflammation of the placental chorion has been linked to neurodevelopmental delays, due possibly to fetal brain and multi-organ injury, as well as to weakened membranes and their premature rupture (PROM) [77]. There also exists a strong correlation between placental inflammation and GDM, which could suggest a disease pathway [78]. Another possible pathway linked to BC-induced adverse health effects, is genotoxicity. Apart from silencing of a gene associated with birth weight [72], there is limited evidence of DNA damage and greater expression of proinflammatory interleukins in bronchoalveolar lavage from intratracheally exposed mice [79,80]. The importance of confirming the highly evocative epidemiological findings with toxicological studies cannot be overstated.

The wider societal and economic impact of prenatal BC exposure is yet to be addressed. For example, the average cost of each preterm birth in the United States—counting medical care, early intervention, and the likelihood of pregnancy loss—is about $65,000 [81]. In 2020, 1 out of every 10 infants born in the U.S. was born preterm [81]. While preterm birth rates seem to be generally declining over time, there are stark maternal and infant health disparities. For example, Black women have the highest rate of maternal mortality and preterm birth in the U.S., followed by Native American, Hispanic, and Asian-American women [82]. This is compounded by the fact that in the U.S., communities of color tend to live on or near food deserts (i.e., low income, urban areas where a significant number of people lives farther than a mile away from the nearest grocery store), industrial facilities, superfund sites, and highways that readily emit high levels of BC and other air pollutants. As a result, women and children in these marginalized communities could be chronically exposed to BC, putting them at comparatively higher risk for developing adverse respiratory, cardiovascular, and reproductive health conditions. The situation is even worse in underdeveloped countries, where there are few environmental standards to regulate emission of air pollutants, and a lack of awareness concerning the potential dangers of BC exposure [83]. Therefore, an urgent call goes out for additional toxicological and epidemiological research on the short- and long-term health effects of prenatal exposure to BC, as well as to policy-makers for appropriate BC standards to be set.

## 4. Future Directions and Gaps in Knowledge

There currently exist no EPA standards for BC which makes regulation and health safety considerations impossible to gauge. More investigations are needed concerning the consequences and implications of BC exposure during pregnancy on both the mother and developing fetus. While information on the effects of prenatal exposure to PM is plentiful and continues to emerge, there is scant reproductive and developmental research performed solely on BC, and the limited data available are mostly of an epidemiological nature, with no attention paid to mechanisms of action. Of the studies cited in this review, some do not include details on BC specifically, and none discuss interactions with other PM constituents as a mixture. To address these knowledge gaps, future research needs to emphasize developmental and reproductive effects of aerosolized BC using animal models, as well as ex vivo and in vitro toxicological studies that can help uncover molecular and cellular mechanisms associated with prenatal BC exposure. Such studies will contribute to a deficient database that can help establish exposure limits for protecting the health of already vulnerable populations. Finally, policy-makers need to acknowledge the potential implications of BC on maternal-fetal health and health-related disparities, as well as for their economic responsibilities to identify solutions and mitigation strategies. Thus, this paper calls for immediate action to protect pregnant women and their unborn children from BC-induced health impacts.

## Figures and Tables

**Table 1:** Summary of prenatal air pollution and black carbon (BC) epidemiological studies (epidemiological, unless otherwise specified).

References	Study Design	Outcome	Contaminant Effect
Fleisch et al., 2015 [13]	Air pollution exposure in the 3rd trimester	Low birth weight	BC↑
Brauer et al., 2008 [14]	Air pollution exposure	Low birth weight; small for gestational age birth	BC↑, PM_10_↑, PM_2.5_↑, CO↑, NOx↑
Rokoff et al., 2018 [15]	Exposure to traffic pollution in combination with smoking and PFAS plasma concentration	Low birth weight for gestational age	BC↑, PFOS↑, smoking↑
Sun et al., 2021 [16]	Air pollution exposure during entire pregnancy period	Decreased Weight-for-Length (WLZ); BMI-for-Age (BMIz); and Weight-for-Age (WAZ) at birth, 1- 4- and 6 years of age	BC↑, PM_2.5_↑, OC↑, NH_4_^+^↑, NO_3_^−^↑, and SO_4_^2−^↑ during the 2nd and 3rd trimesters at 1, 4, and 6 years in boys PM_2.5_↑, NH_4_^+^↑, and NO_3_^−^↑ during the 1st and 2nd trimesters at birth in girls
Dong et al., 2022 [17]	Air pollution exposure	Low birth weight	BC↑, NO_2_↑ during the 2nd and 3rd trimesters at 1,-4, and 6- years-old in boys
Harnung Scholten et al., 2021 [18]	Air pollution exposure in 2nd and 3rd trimester	Decrease in telomere length in umbilical cord leukocytes	BC↑ in the 3rd trimester, but ↓ in the 2nd trimester
Riddell et al., 2022 [19]	Air pollution exposure during pregnancy in Latinx, Black, Asian, and White women	Risk of preterm birth	BC↑, PM_2.5_↑, ozone↑ for Latinx and Black women
Kingsley et al., 2017 [20]	Air pollution exposure	Risk of preterm birth	BC↑
Qiao et al., 2022 [21]	Air pollution exposure	Risk of preterm birth	BC–N.E.
Han et al., 2020 [22]	Air pollution exposure during the 1st trimester	Premature rupture of membranes (PROM)	BC↑
Harris et al., 2015 [23]	Traffic-related air pollution exposure during	Lower verbal and non-verbal IQ; reduced visual motor skills	Living < 50 m to a major roadway↑ BC–N.E.
Harris et al., 2016 [24]	Traffic-related air pollution exposure	Higher executive function	BC↑
Cowell et al., 2015 [25]	Air pollution exposure	Decrease in Attention Concentration Index (ACI)	BC↑, boys > girls
Clark et al., 2010 [26]	Air pollution exposure during pregnancy and the first year of life	Increased asthma incidence	BC↑
Chen et al., 2022 [27]	Air pollution exposure	Increased incidence of hay fever and allergic rhinitis	BC↑, PM_2.5_↑
Chiu et al., 2014 [28]	Air pollution exposure	Childhood wheeze	BC↑, PM_2.5_↑
Witters et al., 2021 [29]	Air pollution exposure in 3rd trimester	Lower skin hyperemia	BC↑, PM_2.5_↑, PM_10_↑ and NO_2_↑
van Rossem et al., 2015 [30]	Air pollution exposure 2 to 30 days before birth	Increase in newborn systolic blood pressure	BC↑, PM_2.5_↑, ozone↓
Madhloum et al., 2019 [31]	Air pollution exposure during the last 4 months of gestation	Increase in newborn systolic and diastolic blood pressure	BC↑
Fleisch et al., 2017 [32]	Air pollution exposure	Increased leptin concentration, total fat mass and insulin resistance upon exposure in the 3rd trimester; Decreased insulin resistance upon exposure in the 1st and 2nd trimester	BC↑
Yu et al., 2020 [33]	Air pollution exposure during 2nd trimester	Increased risk of gestational diabetes mellitus (GDM)	BC↑, PM_2.5_↑
Fleisch et al., 2014 [34]	Air pollution exposure during 2nd trimester	Increased risk of impaired glucose tolerance (IGT)	BC↑, PM_2.5_↑, traffic density↑
Mandakh et al., 2020 [35]	Air pollution exposure	Risk of preeclampsia and small for gestational age birth	BC↑, PM_2.5_↑, PM_10_↑ and NO_x_↑
Gaskins et al., 2020 [36]	Air pollution exposure for women who underwent assisted reproduction	Increased risk of pregnancy loss 30 days after a positive hCG test	NO_2_ ↑ BC–N.E., PM_2.5_–N.E.
Verheyen et al., 2021 [37]	Air pollution exposure in the 2nd and 3rd trimester	Increased hair cortisol concentration	BC↑
Saenen et al., 2016 [38]	Air pollution exposure	Increased 3-nitrotyrosine placental levels	BC↑, PM_2.5_↑
Hargiyanto et al., 2021 [39]	BC exposure of rats via inhalation during pregnancy	Increased amount of Hofbauer cells and expression of NF-κB	BC↑
Neven et al., 2021 [40]	Air pollution exposure	Increased placental iodine uptake	PM_2.5_↑ in the 2nd trimester, but ↓ in the 3rd trimester BC–N.E., NO_2_–N.E.
Howe et al., 2018 [41]	Air pollution during pregnancy	Increased newborn total thyroxine levels (T4)	PM_2.5_↑, PM_10_↑
Neven et al., 2018 [42]	Air pollution exposure	Increased DNA mutation rate and increased methylation of DNA repair genes	BC↑
Kingsley et al., 2017 [43]	Air pollution exposure	Decreased expression of genes associated with fetal growth or cholesterol placental exchange	BC↑

↑—an increase in the air pollutant concentration causes the effect, ↓—a decrease in the air pollutant concentration causes the effect, N.E.—no effect is produced by changes in the air pollutant concentration.

## Data Availability

Not applicable.
